# Supplementary material for: DMRforPairs: identifying Differentially Methylated Regions between unique samples using array based methylation profiles
Source: BMC Bioinformatics. 2014 May 15;15:141. doi: 10.1186/1471-2105-15-141 (PMC4046028; doi:10.1186/1471-2105-15-141)
Supplement: Additional file 2 — DMRforPairs output for the comparison of A431-MCF7 and NA17018-NA17105. Please start from the HTML files in each folder. Available via the BMC Bioinformatics website. [file 1471-2105-15-141-S2.zip › 1394847754114233_MOESM2_ESM/A431_MCF7/figures/27779.pdf]

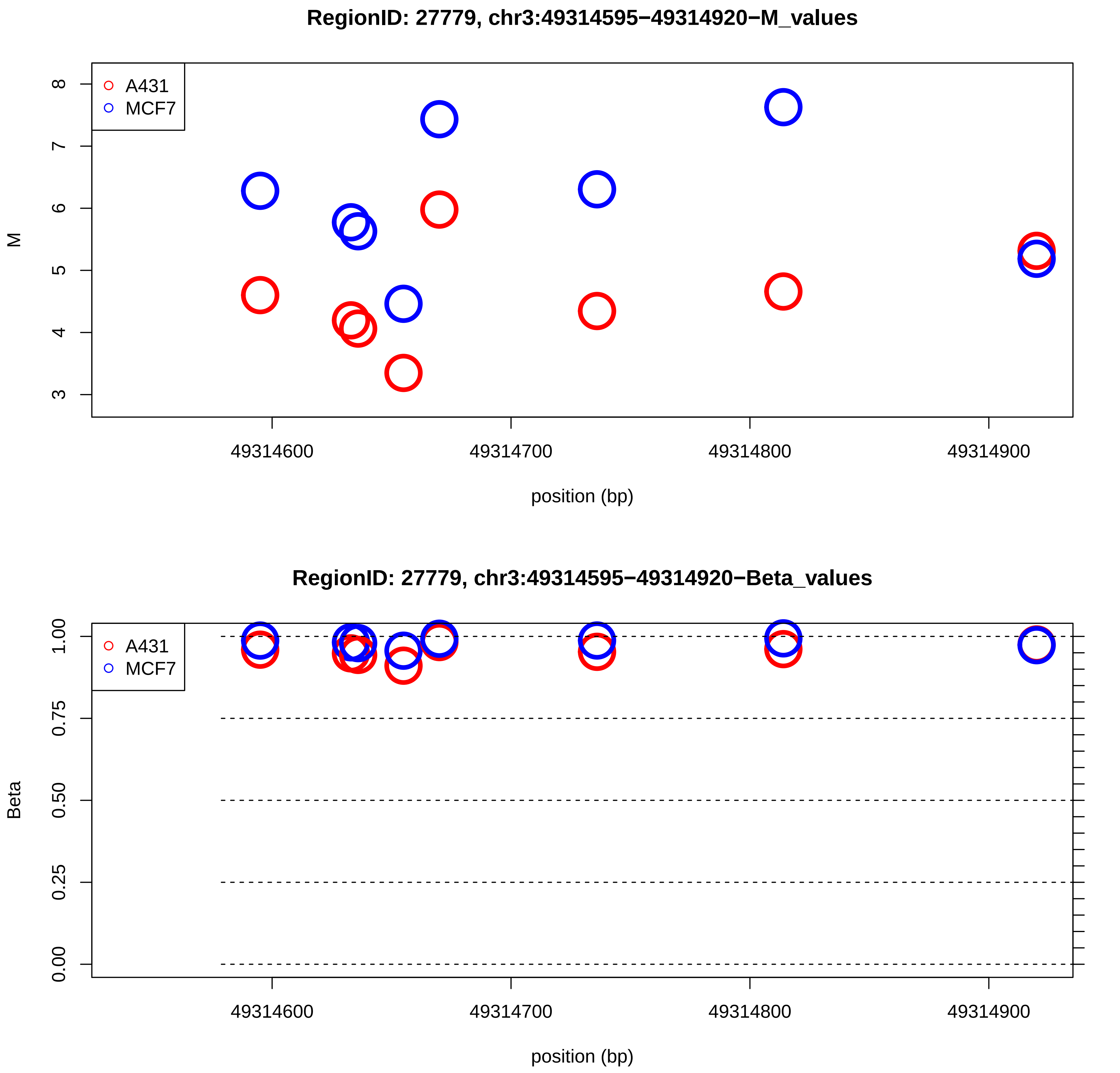

RegionID: 27779, chr3:49314595−49314920−M_values
A431
MCF7
M
3
4
5
6
7
8
49314600
49314700
49314800
49314900
position (bp)
RegionID: 27779, chr3:49314595−49314920−Beta_values
A431
MCF7
Beta
0.00
0.25
0.50
0.75
1.00
49314600
49314700
49314800
49314900
position (bp)

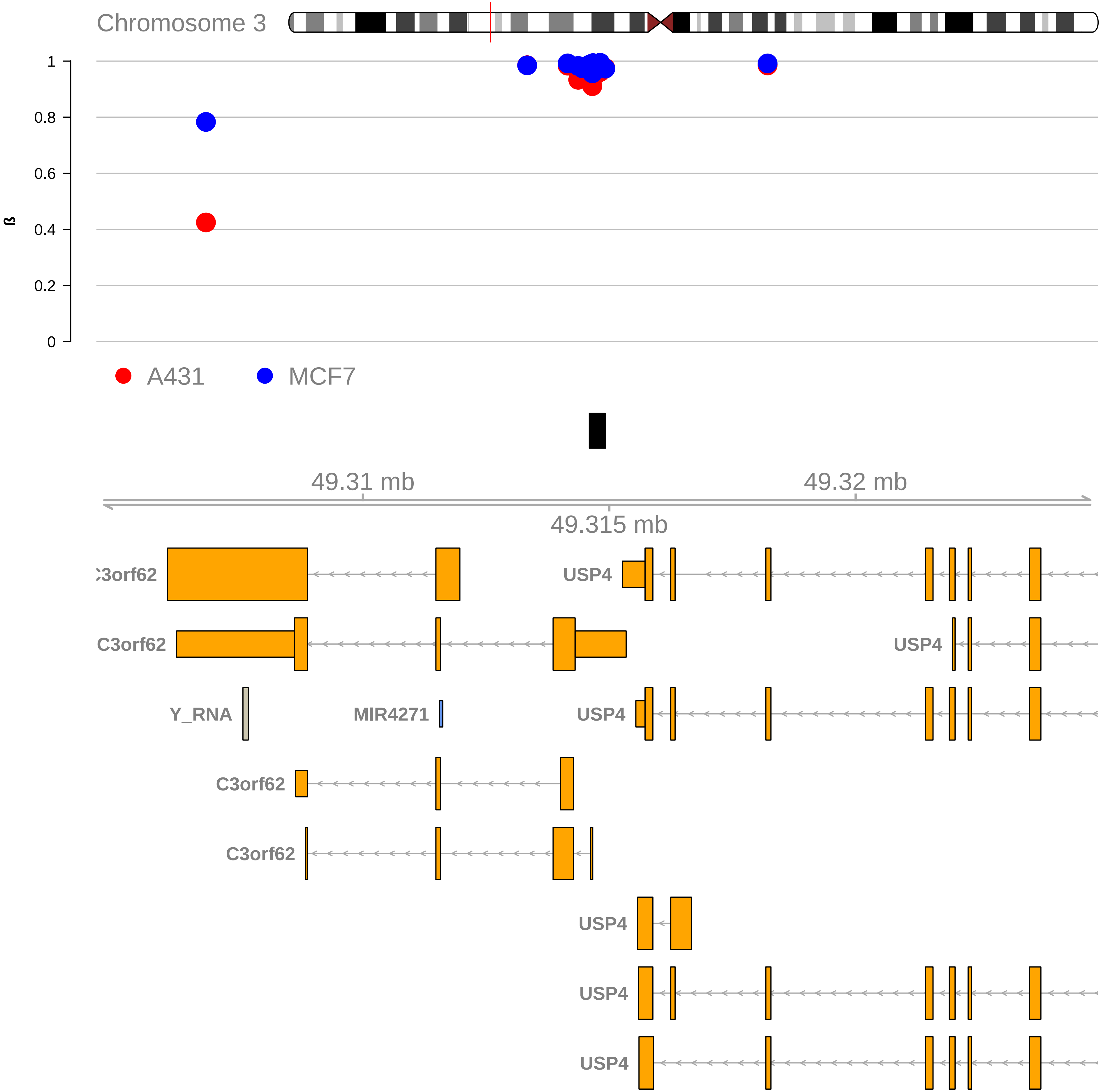

Chromosome 3
1
0.8
0.6
0.4
0.2
0
ß
A431
MCF7
49.31 mb
49.32 mb
49.315 mb
C3orf62
USP4
C3orf62
USP4
Y_RNA
MIR4271
USP4
C3orf62
C3orf62
USP4
USP4
USP4
